# Hybrid Deep Learning Model for EI-MS Spectra Prediction

**DOI:** 10.3390/ijms27031588

**Published:** 2026-02-05

**Authors:** Bartosz Majewski, Marta Łabuda

**Affiliations:** 1 Department of Theoretical Physics and Quantum Information, Gdańsk University of Technology, Narutowicza 11/12, 80-233 Gdańsk, Poland; bartosz.majewski@pg.edu.pl; 2 BioTechMed Center, Gdańsk University of Technology, Narutowicza 11/12, 80-233 Gdańsk, Poland

**Keywords:** electron ionization mass spectrometry, EI-MS spectrum prediction, graph neural networks, deep learning, spectral library augmentation, mass spectrometry databases

## Abstract

Electron ionization (EI) mass spectrometry (MS) is a widely used technique for the compound identification and production of spectra. However, incomplete coverage of reference spectral libraries limits reliable analysis of newly characterized molecules. This study presents a hybrid deep learning model for predicting EI-MS spectra directly from molecular structure. The approach combines a graph neural network encoder with a residual neural network decoder, followed by refinement using cross-attention, bidirectional prediction, and probabilistic, chemistry-informed masks. Trained on the NIST14 EI-MS database (≤500 Da), the model achieves strong library matching performance (Recall@10 ≈ 80.8%) and high spectral similarity. The proposed hybrid GNN (Graph Neural Network)-ResNet (Residual Neural Network) model can generate high-quality synthetic EI-MS spectra to supplement existing libraries, potentially reducing the cost and effort of experimental spectrum acquisition. The obtained results demonstrate the potential of data-driven models to augment EI-MS libraries, while highlighting remaining challenges in generalization and spectral uniqueness.

## 1. Introduction

Mass spectrometry is an important analytical tool for identifying chemical substances and providing indications about their structure and functional groups. A commonly used experimental technique is gas chromatography/electron ionization-mass spectrometry (GC/EI-MS). Particularly, in the EI-MS ionization technique, molecules are bombarded with high-energy electrons (typically 70 eV), causing them to lose an electron and form a radical cation M^+•^. The high energy also generates extensive fragmentation, producing a characteristic pattern of fragment ions. It is a highly reproducible and cost-effective version of MS, and widely used in many fields, such as analytical chemistry, pharmacokinetic studies [1], medicine [2], proteomics [3], as well as metabolomics [4]. With the constant increase in the instrumentation development, the popularity and broad applicability of GC/EI-MS have caused sustained growth in the size of spectral databases. Experimentally acquired spectra can be queried against such databases to identify compounds and infer molecular structural properties [5,6]. Despite continuous expansion, database coverage remains limited because many compounds have not yet had their spectra measured; the absence of a reference spectrum for a target compound can therefore lead to misidentification. This creates a need for commercial spectral databases to regularly release updates. Consequently, commercial spectral libraries require periodic updates, a process that depends on the time and resources available for acquiring new spectra. For example, one that is widely used is the National Institute of Standards and Technology (NIST) Mass Spectra Library [7], adding roughly only 40,000 spectra to its EI-MS reference collection every three years. However, these updates tend to favor molecules of broad interest, so newly synthesized or less-studied compounds are often absent or underrepresented. Augmenting existing reference libraries with synthetic spectra generated by computational models is a promising approach to mitigate these limitations.

EI-MS spectrometry remains one of the most widely used analytical techniques for structural characterization, yet accurately predicting EI mass spectra from first principles is challenging because the process involves ionization and multi-step fragmentation of the ions [8]. Over the years, multiple computational approaches have been developed to address different aspects of the problem. Reaction-network and kinetics-based approaches (e.g., Rice–Ramsperger–Kassel–Marcus (RRKM) theory) approximate fragment abundances by mapping stationary points on the potential-energy surface and evaluating microcanonical rate constants [9]. High-level ab initio wavefunction methods such as Equation-of-Motion Ionization Potential Coupled Cluster (EOM-IP-CCSD) provide accurate ionization energies and electronic-state characterization, forming the foundation for modeling the initial ionization event [10]. Fragmentation can be simulated either through ab initio or semi-empirical molecular dynamics such as Born–Oppenheimer molecular dynamics (BOMD) or semi-empirical tight binding methods (e.g., GFN-xTB), which dynamically explore bond dissociations and rearrangements under EI-like energy conditions [8]. Collectively, these approaches offer a spectrum ranging from high-accuracy to high-throughput methods, enabling the increasingly realistic and systematic prediction of EI mass spectra.

RRKM theories explicitly model the redistribution of energy over the internal degrees of freedom and allow the estimation of the rate constants for the ionization reaction [11,12,13,14]. To apply this theory, we assume an ensemble of molecules that have been excited to a state in which they carry a fixed amount of energy *E*, a portion of which is in rotational motion, while the rest is stored in vibrational modes. According to RRKM theory, the number of possible reaction pathways is determined by the density/sum of the vibrational states. As the number of vibrational modes increases, the number of possible states grows rapidly. This limits the application of such theories to small molecules, as the count of vibrational modes increases with the size of the molecule [9].

Another line of predicting mass spectral fragmentations is to directly predict where fragmentation may occur within the molecule. There are approaches which do not use statistical theory and instead perform quantum chemical calculations to map stationary points on the potential-energy surface (PES). By examining reaction energies and molecular structures, these methods allow for the qualitative prediction of the primary decomposition pathways for molecular ions. There have been approaches that declared that EI mass spectra of simple organic compounds could be predicted through calculations of bond orders and partitions of Hamiltonians as in the work of Mayer and co-workers [15,16]. Additional approaches rely on the Binary-Encounter Bethe (BEB) formalism to map the deposition of excess energy in a molecule and to compute the corresponding internal energy distribution of the molecular ions [17].

On the other hand, methods based on BOMD allow for modeling the time evolution of the fragmentation event. In this approach, an EI-induced fragmentation is simulated by propagating nuclear motion on an electronic potential surface that is recalculated at each timestep. In this framework, the fragmentation event is represented by preparing the neutral molecule with appropriate excess energy. Due to the presence of excess energy, the system naturally explores possible bond cleavages and rearrangements that are consistent with underlying quantum-mechanical forces. A relatively recent example of ab initio is the QCxMS, a quantum chemical (QC)-based program that enables users to calculate mass spectra using Born–Oppenheimer molecular dynamics. The related software is developed by the group of S. Grimme [8,18,19]. Such methods enable the statistical sampling of molecular-dynamics trajectories. In consequence, multiple trajectories yield a distribution of possible fragments. Aggregation and renormalization of the fragments across all trajectories produces a simulated EI-MS spectra. The main limitations of these methods arise from their high computational cost. Because BOMD evaluates energies and gradients at a chosen electronic-structure level (one of the most time-efficient approaches with acceptable accuracy is the Density Functional Theory (DFT); however, in principle it usually is a higher level method, such as the post–Hartree–Fock methods: MP2 or CCSD), it is computationally demanding, requiring many long trajectories for statistical convergence, and may still struggle to capture rare events or non-adiabatic effects relevant to certain EI processes. Even with access to high-performance computing units, simulations may require several hours to generate a single trajectory for a medium-sized molecule. Such time constraints prevent the rapid generation of large numbers of spectra. Moreover, although these methods can often identify potential fragmentation pathways, their overall accuracies are frequently insufficient for reliable compound identification [20].

Data-driven methods for MS have been explored for a long time. Interest in computational models for predicting and identifying compounds from their mass spectra dates back to the 1960s with the DENDRAL project, which began in 1965 and produced the first well-documented expert system for assisting organic chemists in structure elucidation from mass spectra [21,22]. Since then, many algorithms and machine learning models have been developed for a range of MS-related tasks, including spectrum prediction, peak annotation, and spectral library searching [23,24,25,26,27,28,29].

Several models have been specifically designed for the direct prediction of EI-MS spectra. Competitive fragmentation modeling (the CFM family) introduced a probabilistic, rule-informed fragmentation model and has been developed both for tandem MS (CFM-ID) and for EI spectra (CFM-EI) [30,31,32,33,34]. CFM models use a tree-based probabilistic generative architecture, where the fragmentation of a molecule is modeled as a fragmentation tree that recursively represents how a parent molecule breaks into smaller pieces, predicting both mass/charge values and intensities. Notable examples of neural approaches for EI-MS prediction include NEIMS (Neural Electron–Ionization Mass Spectrometry) and RASSP (Rapid Approximate Subset-based Spectra Prediction). NEIMS uses extended-connectivity fingerprints as input to a feed-forward network with a bidirectional prediction mode that is the most characteristic feature of the NEIMS’ architecture [35]. The bidirectional prediction combines forward and reverse predictions to better capture neutral fragment losses. RASSP combines substructure enumeration with graph-based deep learning [36]. The RASSP model architecture is much more sophisticated than NEIMS; model preparation employs extensive subformulae and a subset generation algorithm in order to gather additional training data. It is divided into two independent models, one based on chemical formulas and the second on subsets of molecular fragments. It uses a common graph neural network encoder which learns atom representations which are later used for predicting a probability distribution over atom subsets and chemical subformulae. The resulting spectra are a weighted sum of probability-based intensities of given atom subsets and subformulae.

Many molecule-related problems have been addressed using graph neural networks [37,38], for example to create molecular representations [39,40] instead of relying on handcrafted fingerprints such as Morgan’s fingerprints [41]. This strategy is implemented, for example, in RASSP, as well as in models that aim to improve the NEIMS architecture [42], and others [43,44,45,46]. It has been shown that GNN architectures are capable of learning molecular representations that are often competitive with handcrafted ones, while substantially reducing the dimensionality of learned representations compared to fingerprints such as Morgan’s [47,48,49,50,51]. Handcrafted fingerprints also face the issue of bit collision due to the limited number of available bits. Increasing fingerprint dimensionality can reduce collisions, but learned dense embeddings, such as those produced by GNNs or other machine learning models, overcome this problem by allowing each substructure, atom, or bond to contribute directly to a real-valued representation rather than being forced into a fixed bit slot [52]. Recent GNN-based studies in drug–biological system modeling and mechanistic prediction have demonstrated the effectiveness of tailored message-passing schemes and graph-level embeddings for capturing complex, structured biochemical interactions [53,54], providing a broader perspective for the design of our GNN encoder and its application to modeling EI-MS fragmentation behavior.

The currently available models struggle with rare or complex fragmentation patterns and are often biased due to the underrepresentation of certain chemical classes in widely used spectral libraries. Moreover, most models with non-trivial architectures are closed solutions, making direct extension difficult or inefficient. In this work, we present a model that exploits the molecular representation learning capabilities of GNNs and leverages a ResNet-based decoder to accurately map these learned embeddings to predicted the EI-MS spectra, capturing complex nonlinear relationships between molecular structure and fragment intensities. Furthermore, in contrast to previous studies, the proposed model incorporates a dedicated refinement stage that explicitly injects physical and chemical knowledge through mass- and neutral-fragment-guided masks. This refinement framework is inherently modular and can be extended with additional mask-based components to further enhance the fidelity of the final predicted spectra. The model additionally integrates a cross-attention mechanism and a bidirectional prediction scheme inspired by NEIMS [35]. The flexibility of the refinement stage enables the accommodation of edge and exotic cases, including unusual fragmentation pathways or rare chemical motifs, by incorporating supplementary mask-based knowledge. In addition, we adopt a modified Recall@k evaluation strategy based on spectral embeddings. Specifically, our approach leverages spectral projections of both true and predicted spectra, which are integrated into the model training process through the inclusion of the retrieval-based loss component in the objective function. This formulation promotes greater uniqueness in the generated spectra, leading to improved Recall@k performance. Unlike prior EI-MS prediction studies, we conduct systematic ablation studies across multiple random seeds to assess model robustness and quantify the contribution of individual architectural components. Moreover, we provide a more detailed characterization of model performance by analyzing results across different molecular-weight bins. Finally, we provide quantitative measures of chemical diversity within the employed datasets by assigning molecules to coarse molecular superclasses and chemical classes.

## 2. Results and Discussion

Training pipeline. To examine the performance of the model, we used the NIST14 EI-MS database. During training, the main library (mainlib) was used. It was ensured that the molecules present in the replicate library (replib) had been removed from the mainlib before the model was trained to prevent information leakage. With removal of replicate molecules and enforcing SMILES (Simplified Molecular Input Line Entry System) uniqueness, the main library dataset amounted to 201,187 molecule–spectra pairs. The dataset was divided into training and test sets with 80/20 ratio. The performance of the model was analyzed across ten different random seeds to quantify its statistical variability, with the following random generators set:

random.seed(seed)
np.random.seed(seed)
torch.manual_seed(seed)
torch.cuda.manual_seed_all(seed)
torch.backends.cudnn.deterministic = True
torch.backends.cudnn.benchmark = False

Dataset splits were obtained using a separate seed value of 42, with the same split used in all ten experiments for which the performance was tested. However, during training, the shuffling of the training set was enabled. The above approach was also used to perform ablation studies of the main components of the model, to determine their importance in operation of the model. Ablation studies consisted of checking the following model variants:

disable_attention—baseline model with disabled cross-attention,
replace_resnet_with_linear—baseline model with ResNet replaced by a simple linear neural network,
fix_alpha_forward—baseline model with only forward prediction, reverse prediction disabled in bidirectional prediction mode,
replace_gnn_with_pool_mlp—baseline model with MPNN (Message Passing Neural Network) encoder replaced by simple aggregator and MLP (Multi-Layer Perceptron),
disable_mask—baseline model with learnable probabilistic mask disabled.

The average values of the chosen metrics, together with their standard deviations, for all ten seeds are given in Table 1.

**Figure 1:**
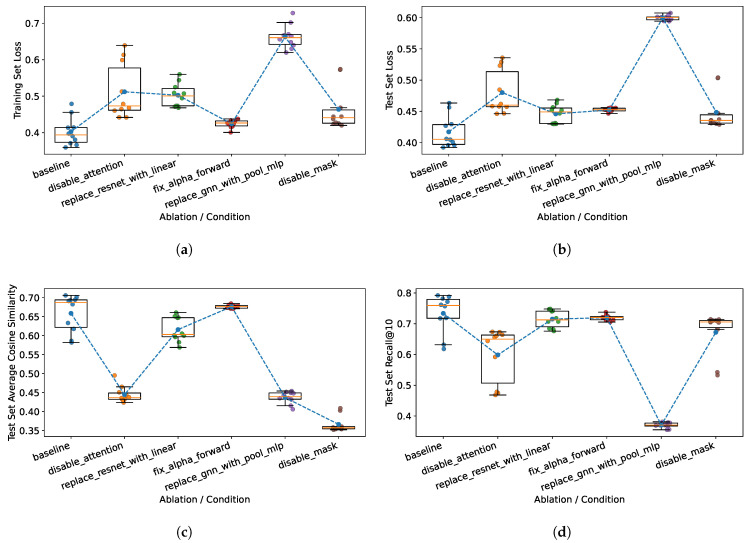
Box plots of the metrics across all chosen random seeds and model variations during ablation studies. (**a**) Distribution of loss values during training for the training set. (**b**) Distribution of loss values during training for the test set. (**c**) Distribution of average cosine similarity values during training for the test set. (**d**) Distribution of Recall@10 values during training for the test set.

A more detailed distribution of the most important metrics is presented in Figure 1. The results indicate that the baseline model achieves the best performance within the statistical variability. It means that all the components verified through the ablation studies contribute positively to the final architecture. Furthermore, the box plots in Figure 1 reveal that the bidirectional prediction module is the main source of instability, as removing the reverse-prediction pathway reduces the standard deviation of all evaluated metrics across random seeds. The probabilistic mask exhibits similar behavior, as only individual random seeds deviate substantially from the average metric values. However, it should be noted that the two modules do not influence the model in exactly the same way. This is proven by the apparent difference in similarity values presented in Figure 1c. The third ablated component that reduces the variance of the metrics is the MPNN encoder. However, in contrast to bidirectional prediction and the probabilistic mask, this reduction is attributable to a substantial degradation in model performance. Replacing the MPNN encoder with a simpler MLP restricts the model’s learning capacity, resulting in consistently poor metric values across all random seeds. While the MPNN encoder is the most important overall, cross-attention and the probabilistic mask also show high importance in achieving a high cosine similarity between predicted and measured spectra (Figure 1c). This is an interesting result since one might assume that a strong influence on cosine similarity values would directly affect the Recall@k values. Figure 1d shows that this is not the case, as ablating the cross-attention and probabilistic mask modules only marginally degrades the Recall@10 values. These results suggest that high cosine similarity is not a strong determinant of Recall@k performance and provide evidence for a partial decoupling between spectral reconstruction quality and retrieval performance. This indicates that these modules primarily improve fine-grained spectral fidelity rather than the discriminative structure of the embedding space used for library matching, and that the retrieval head is robust to spectral imperfections that strongly impact cosine similarity.

**Figure 2:**
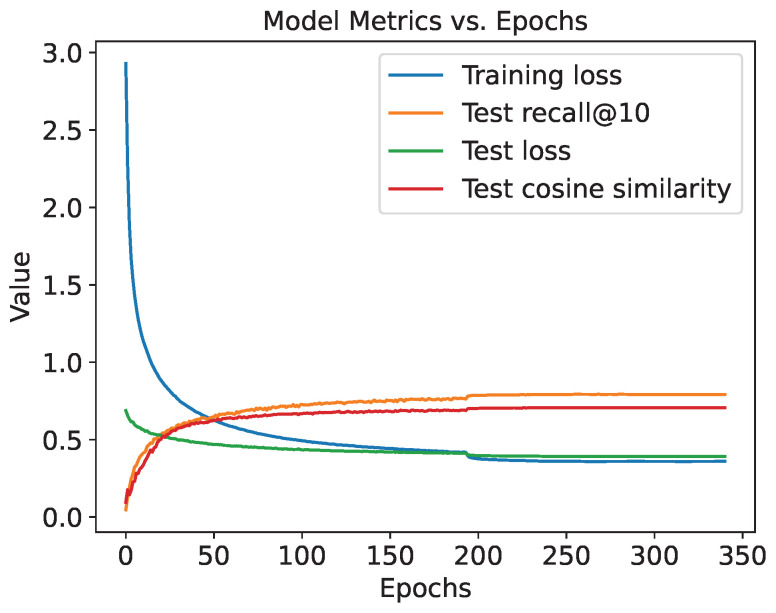
The training dynamics of the seed chosen for the library matching task.

From the ten random seeds used to evaluate the baseline model, the seed yielding the highest Recall@10 value was selected for subsequent, detailed performance analysis. The training dynamics of the chosen seed are shown in Figure 2.

Library matching task. The next stage of evaluation involved assessing the spectra predicted by the trained model within a library matching framework. The protocol used to quantify the matching accuracy of the predictions followed the procedures described in [35,42].

The predicted spectra for the molecules present in the replicate library were used to create an augmented spectral library by adding them to the main library. Following the reduction of the replicate library to retain only unique SMILES, 20,265 samples remained, yielding an augmented library containing 221,452 entries. The ground truth spectra of replib molecules were used as queries. Each query was compared with the augmented library, and their pairwise similarity was computed. The highest k similarity values for k∈{1,5,10} were saved for each query molecule.

In contrast to previous studies, in our model, similarity was not computed directly between the raw spectra and their predicted counterparts. Instead, comparisons were performed in the learned embeddings using the outputs of the projection head. The similarity measure used to compute similarities between the embeddings was quantified using the standard dot-product. In this case, all embeddings were L2-normalized; therefore, the dot-product reduces to simple cosine similarity. Moreover, during the retrieval of the top-k values and the evaluation of Recall@k, a mass filter was used, following the schemes adopted in refs. [35,36]. In the calculation of Recall@k, the filter considers only molecules of mass within ±5 Da of the mass of the query molecule. The model achieved following Recall@k values for the library matching task: 80.8% Recall@10, 67% Recall@5, and 25% Recall@1.

The Recall@10 and Recall@5 values in the library matching task are close to the levels achieved by other EI-MS prediction models. Our Recall@10 and Recall@5 results differ from previous models by 5.9–12.7% from the declared values. However, the decline in Recall@1 is substantially more pronounced than in comparable models. This indicates, that the spectra generated by the proposed model may lack sufficient uniqueness and that its capacity for generalization may be limited -both factors contribute to the final result. Molecules in the replicate library exhibit limited structural similarity to those in the training set, a trend observed across multiple versions of the NIST EI-MS library [35,36,42]. We verified it for NIST14 by Tanimoto similarity calculations and found that only 23.6% of replib molecules have a counterpart in mainlib with a similarity of 0.8 or higher. The bad generalization assumption would mean that the model learned to predict spectra that capture structural dependencies of molecules with structures mostly similar to those present in the training set. This would partially explain why Recall@1 is worse in library matching with replicate library than during the training, while Recall@10 and Recall@5 are very similar. The model proposed in this work shares similar core components as the model presented in [42]: GNN encoder, ResNet decoder and bidirectional prediction. Our model additionally incorporates the cross-attention and probabilistic mask mechanisms, as well as a recall-oriented loss function. Consequently, the two models are expected to exhibit at least comparable performance. This expectation is largely confirmed by the key performance metrics, which differ only by a few percentage points. Our model achieves the Recall@10 and Recall@5 values lower by 5.9% and 12.1%, respectively. Moreover, it is important to emphasize that our model was trained on the NIST14 library, which exhibits substantially greater chemical diversity than the NIST05 library used by Zhang and co-workers [42]. This discrepancy is reflected, for instance, in the size of the atomic number one-hot encoding: the NIST14 database contains 17 additional atom types present in the molecular spectra. The higher molecular diversity in NIST14 increases the complexity of the learning procedure and makes achieving the same level of generalization more challenging.

Limited spectral uniqueness may also contribute to the reduced performance. This concern partially motivated the inclusion of a retrieval loss component to the total loss function and the incorporation of a projection head into the model. During the development of the model, we observed that training only against an MSE-type loss caused the model to predict spectra that exhibited high similarity scores with many candidate molecules simultaneously. Because the model cannot produce perfectly accurate spectra, those distractor spectra could have been chosen over the spectra for the specific molecule for which the prediction was made. This behavior suggests that either (i) the representations of molecules produced by the MPNN encoder were insufficiently separated in latent space, or (ii) the structural model components such as the learnable probability mask, intended to encode group-specific fragmentation characteristics, may have induced excessive similarity across the predicted spectra.

The incorporation of a cross-entropy-based loss function, a projection head, and expanded one-hot feature encodings was designed to maximize the separation of molecular embeddings within the representation space. This objective was successfully achieved since these modifications proved effective, yielding substantial improvements in recall performance. Specifically, the highest Recall@10 observed prior to introducing the retrieval loss was approximately 45%. It is also worth noting that applying a mass filter during the Recall@k evaluation produced further improvements, increasing Recall@k by 10–25% depending on the random seed and the value of k.

**Figure 3:**
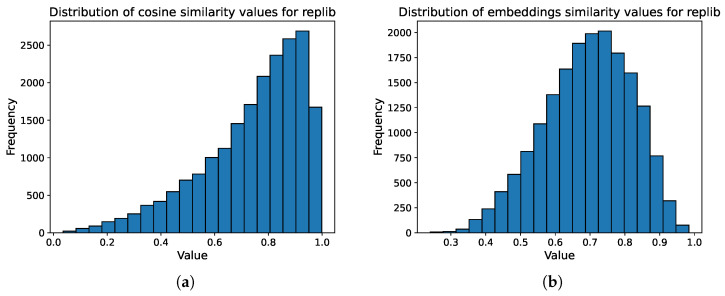
Distributions of similarity values, (**a**) cosine similarity between raw predicted and ground truth spectra, (**b**) cosine similarity between projection head embeddings of predicted and ground truth spectra.

Average similarities. Additionally, we analyzed the distribution of cosine similarity values between the ground truth and predicted spectra for the replicate library, considering both the raw and embedded spectra (Figure 3). For raw spectra, the average cosine similarity was 0.74, with approximately 79% of values exceeding the 0.6 threshold, which is almost on par with the results achieved by [42]. For the embedded spectra, the average similarity was 0.69, with about 77% of the values above 0.6. From the cosine similarity value distributions, it is evident that although the average is high there are still many spectra with low similarity scores. This once again implies that the model is capable of creating spectra that on average agree with the training data but struggles with examples that deviate from said average. An interesting result is that despite the lower average similarity values of the embeddings, the scheme using them achieves drastically better recall values, which validates their use, as well as suggesting that higher separation of data points in the representation space at the expense of lower average similarities might be beneficial when it comes to Recall@k values in library matching tasks.

Models comparison. To further validate the performance of the proposed model, we compared it with two pretrained NEIMS and RASSP SubsetNet models, available on their respective public repositories. Firstly, it should be noted that SubsetNet cannot predict spectra for the entire replib because it is restricted to molecules composed only of the elements {H, C, O, N, F, S, P, Cl} and by limitation in subset enumeration [36]. As a result, SubsetNet is unable to generate predictions for the full replicate library, reducing the applicable subset to 17,357 molecules. We refer to this as “RASSP split replib” (RS replib).

To ensure a fair comparison, both the proposed model and NEIMS were evaluated on the full replib as well as on the RS replib, whereas SubsetNet was evaluated exclusively on the RS replib. For all models, the predicted spectra were generated using the corresponding pretrained networks and subsequently incorporated into the library-matching task following the same evaluation protocol as applied to the proposed model. Performance was quantified using Recall@k metrics, and average cosine similarity was additionally reported. To provide a more detailed analysis, Recall@k values were computed separately for three molecular-weight bins: small (1–175 Da), medium (176–324 Da), and large (325–500 Da). A summary of all the results is presented in Table 2, where the proposed approach is denoted as HYBRID.

SubsetNet achieves the highest overall performance among the evaluated methods, with the exception of the largest molecular-weight bin. The proposed HYBRID model consistently surpasses NEIMS on nearly all Recall@k metrics, the only deviation being Recall@1 for large molecules. It also exhibits a modestly lower average cosine similarity across the considered mass ranges. It is important to emphasize that SubsetNet is trained on a smaller and more chemically restricted dataset, excluding inorganic species and limiting overall chemical diversity. Moreover, SubsetNet employs a substantially larger architecture and relies on a computationally expensive bond-breaking-based subset enumeration procedure, which increases inference time and limits scalability for large libraries. In contrast, the proposed HYBRID model attains competitive retrieval performance while maintaining a more compact architecture, faster inference, and broader chemical coverage, including support for inorganic atoms. These characteristics render HYBRID a more versatile and scalable solution for large-scale EI-MS library augmentation, despite SubsetNet achieving higher performance within its narrower applicability domain.

It should also be noted that EI-MS prediction models are known to be sensitive to experimental setup and spectral comparison methodology [55]. The evaluation protocol employed in this work differs from that used in the original NEIMS study [35], which likely contributes to the observed reduction in NEIMS performance relative to previously reported values. Notably, a similar decrease in Recall@1 performance is observed for both NEIMS and HYBRID. While this may suggest an influence of the spectral projection-based retrieval scheme, this effect is not observed for SubsetNet, indicating that the reduced Recall@1 performance in NEIMS and HYBRID cannot be attributed solely to the retrieval formulation. Instead, it likely reflects broader factors, including increased chemical diversity and the inclusion of inorganic species. A comprehensive assessment of these effects would require retraining HYBRID and NEIMS on SubsetNet’s restricted dataset and recomputing all evaluation metrics; however, this dataset is not publicly available, and such an analysis is therefore beyond the scope of the present study.

**Figure 4:**
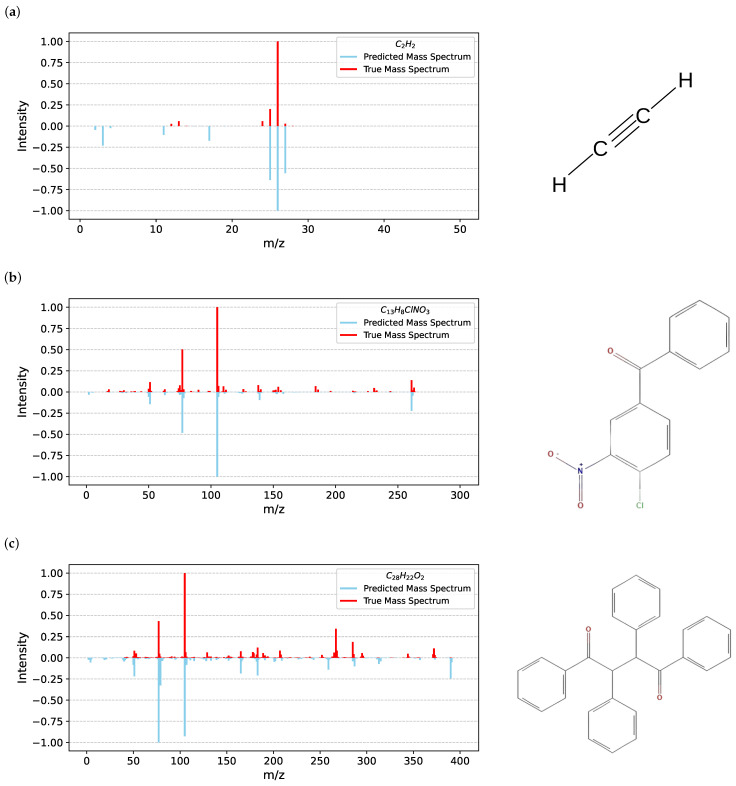
Examples of the predicted spectra for chosen molecules and their chemical structures. (**a**) True vs. Predicted Mass Spectrum for $C_{2}H_{2}$. (**b**) True vs. Predicted Mass Spectrum for $C_{13}H_{8}{\mathrm{ClNO}}_{3}$. (**c**) True vs. Predicted Mass Spectrum for $C_{28}H_{22}O_{2}$. Cosine similarities: (**a**) 0.83, (**b**) 0.98, (**c**) 0.8.

Examples of the spectra predicted by HYBRID for different values of cosine similarities are depicted in Figure 4 for the chosen molecules with the different size and chemical properties. Acetylene $(C_{2}H_{2}),$ 4-chloro-3-nitrobenzophenone $(C_{13}H_{8}{\mathrm{ClNO}}_{3}$) and 1,2,3,4-tetraphenyl-1,4-butanedione $(C_{28}H_{22}O_{2}$) were chosen to validate the efficiency of the hybrid model. In 2 out of 3 of the presented spectra examples the most intensive peak is placed correctly, with the third example having two of the top peaks switched. Overall peak placement is more accurate for heavier molecules; the intensities of the peaks differ from the measured mass spectra, but they mostly retain their proportions in terms of order from highest to lowest intensities.

**Figure 5:**
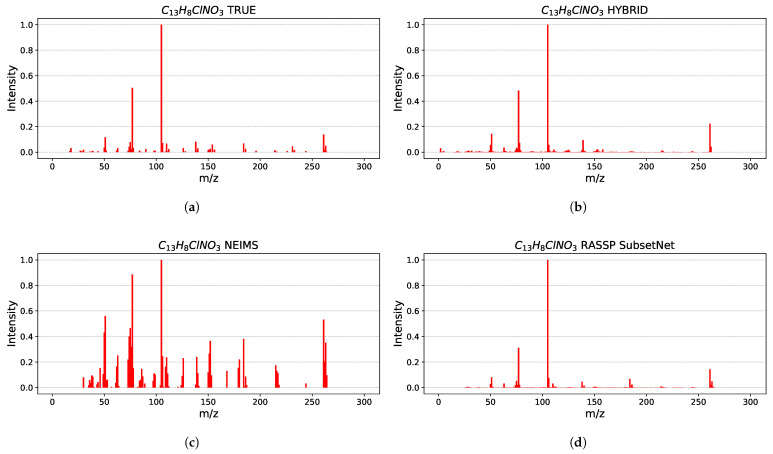
Comparison of 4-Chloro-3-nitrobenzophenone $C_{13}H_{8}{\mathrm{ClNO}}_{3}$ EI-MS predictions with experimental data. (**a**) Experimental spectrum from NIST14 database. (**b**) Spectrum predicted by HYBRID. (**c**) Spectrum predicted by NEIMS. (**d**) Spectrum predicted by RASSP SubsetNet.

Moreover, in Figure 5 we present the spectra predicted by the three compared models alongside the experimental spectrum for the molecule with the highest similarity value in Figure 4. The spectrum predicted by NEIMS immediately stands out: it contains many more visible peaks, and although the locations of the most intense peaks are mostly correct, their intensities deviate substantially from the experimental values. Further inspection of other NEIMS predictions reveals this to be a recurring pattern; its spectra are typically much busier than their experimental counterparts.

In contrast, the predictions of HYBRID and RASSP SubsetNet are much closer to the experimental spectrum. The positions of the main peaks are accurate, and their intensities are reproduced more faithfully than in NEIMS. The HYBRID model better captures the intensity of the secondary peak, although it slightly overestimates the tertiary peak. RASSP SubsetNet shows the opposite behavior: it estimates the tertiary peak well but underestimates the secondary one.

This comparison also partially illustrates why cosine similarity alone is not a sufficient metric. Both HYBRID and SubsetNet achieve a cosine similarity of 0.98 with the experimental spectrum, whereas NEIMS reaches only 0.75. One might expect a 75% agreement to produce a spectrum closer to the experimental one than that shown in Figure 5c. However, this example—near the average similarity of NEIMS—highlights the limitations of relying solely on cosine similarity.

Based on the comparative results, the HYBRID framework demonstrates the ability to partially alleviate the coverage limitations of existing reference mass spectral libraries.

## 3. Materials and Methods

**Figure 6:**
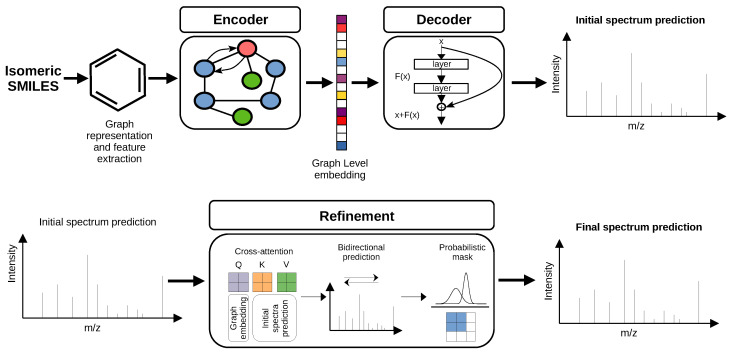
The overall architecture of the proposed model and overview of the framework.

We propose a hybrid deep learning model architecture that can be divided into three main compartments—encoder, decoder, and refinement—as presented in Figure 6. It shows a step-by-step procedure, starting from conversion of isomeric SMILES strings to graphs which are fed into the GNN encoder. The encoder learns effective molecule representations, which are later decoded by a residual neural network. The spectra proposed by ResNet are further refined by cross-attention, probabilistic mask and bidirectional prediction. The primary aim of the model is to provide accurate EI-MS predictions for molecules in the 1–500 Da range, that can be used to augment existing spectral libraries. Furthermore, the main advantage of the structure of this model is that its architecture was intentionally designed with the objective of supporting possible additional mask-like modules. Those structures aim to improve model’s performance within the chosen data distribution or generalization to out-of-distribution examples.

### 3.1. Feature Extraction

The sole input to the model is isomeric SMILES strings. Using the RDKit 2024.03.6 package [56], they are processed into graphs, from which simple atom and edge features are extracted using basic package functionalities. Created graphs do not contain nodes representing hydrogen atoms; we use the number of hydrogens attached to each node as an atom feature. The atom and edge features used are presented in Table 3. One-hot encodings are used to represent the features to separate the embeddings of molecules of similar structure as much as possible. The features used for the atoms and bonds are almost the same as in [42], with the difference that we used explicit valence as can be seen in Table 3.

### 3.2. Datasets

During the development of the model, the NIST14 spectral database was used. The NIST EI-MS spectral database contains two separate libraries: main and replicates library. From the EI-MS fraction we selected spectra of molecules with mass equal or less than 500 Da. The mass was limited to 500 Da because most of the molecules present in the library are lighter than that, making heavier molecules underrepresented, which could have significantly worsened the performance of the model, especially because accommodation of all molecules present in the dataset would have required expanding the dimension of predicted spectra from 500 to over 1600, making most entries even more sparse and causing signal dilution. Since the database does not contain SMILES strings, they were collected by querying the PubChem database [57] with InChIkeys present in the NIST14 EI database. Isomeric SMILES can be ambiguous, that is, the same SMILES may represent molecules with identical graphs but different exact 3D coordinates. The exact coordinates influence specific fragmentation patterns, and although NIST14 provides 3D coordinates, the geometry of each molecule during spectrum measurement may differ. This is why we did not use the 3D coordinates as features. While the geometries are optimized, NIST cautions against using them for critical applications. We consider representing geometries via SMILES sufficient and a more robust solution. To avoid duplication issues, only the first occurrence of each SMILES is retained in the final datasets.

**Figure 7:**
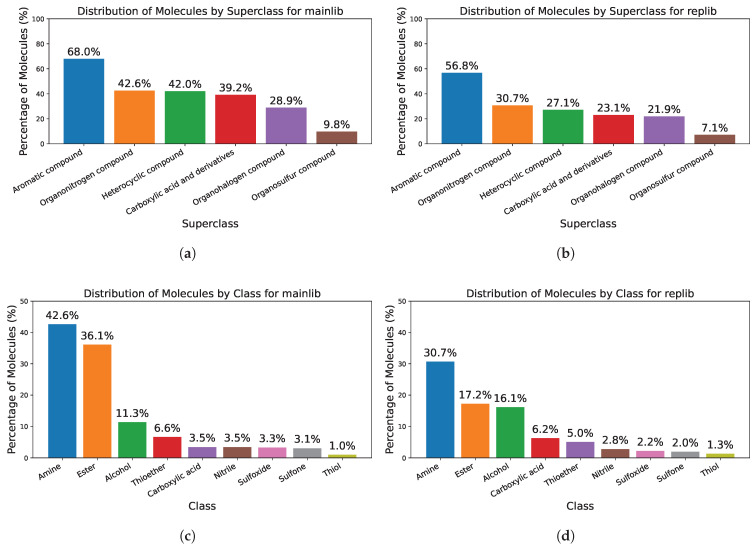
Distribution of NIST14 EI spectral library molecules into superclasses and classes. (**a**) Superclass distribution for mainlib. (**b**) Superclass distribution for replib. (**c**) Class distribution for mainlib. (**d**) Class distribution for replib.

We analyzed the chemical diversity of the libraries with a simple algorithm that leverages the RDKit substructure-matching capabilities [56]. A curated set of SMARTS (SMILES Arbitrary Target Specification) patterns was used to detect functional groups, and molecules were grouped according to a coarse-grained version of the classification schemes in [58,59]. Figure 7 shows the percentage of molecules assigned to each superclass and class for both mainlib and replib. Moreover, we computed the Tanimoto similarity of ECFP4 fingerprints between replib and mainlib. Only 23.6% of replib molecules have a counterpart in mainlib with a similarity of 0.8 or higher.

### 3.3. Model’s Architecture

#### 3.3.1. GNN Encoder

In the first stage, the model uses a graph neural network to learn molecular representations, encoding each molecule’s structure into a graph-level embedding. The graph encoder is based on the PyTorch Geometric 2.6.1 implementation of the MPNN [60]. Message passing in MPNN can be described as

(1) $$x_{i}^{\left(k\right)}=\gamma^{\left(k\right)}\left(x_{i}^{\left(k-1\right)},\underset{j\in N_{i}}{⨁}\phi^{\left(k\right)}\left(x_{i}^{\left(k-1\right)},x_{j}^{\left(k-1\right)},e_{j,i}\right)\right)$$

where ⊕ denotes a differentiable, permutation invariant function; here, it is a sum. γ and ϕ denote differentiable functions; in this case they are MLPs.

#### 3.3.2. ResNet Decoder

The ResNet decoder is based on a shallow residual network architecture. It takes the output of the graph encoder as input and produces initial, unrefined spectra. It is made of one ResNet block and three projection layers that scale up the block output to 512 dimensions, then reduce it to 256 and finally give an initial prediction of dimension 500. Upscaling increases the feature space so the network can represent more complex patterns. The downscaling that follows compresses the rich features into a more compact, informative representation. This helps the model focus on the most important signals and reduces overfitting. Between each linear layer, we used layer normalization. Hyperparameters of ResNet block and other parts of the model can be seen in Table 4. Spectra are predicted at integer resolution, where each index in the predicted output corresponds to an integer m/z value, with the assumption that index 0 corresponds to *m*/*z* = 1.

#### 3.3.3. Initial Prediction Refinement

Cross-attention. Refinement of the initially predicted spectra begins with a cross-attention module that dynamically reweights spectral regions according to the molecular context, thereby emphasizing chemically meaningful peaks. In this module, the graph-encoder output serves as the query, whereas the keys and values are derived from the initial spectral prediction. The dimensions of queries, keys, and values are matched by projecting all three to the same dimension using linear layers (this dimension is equal to the dimension of graph encoder output). This mechanism enables the model to adaptively highlight fragmentation patterns that are most consistent with the underlying molecular structure.

Bidirectional prediction. Following the cross-attention module, a modified bidirectional prediction scheme based on the one introduced by the NEIMS model [35]—is applied. In contrast to the original formulation, we omit the small mass-shift that allows fragment peaks to exceed the molecular mass due to isotopic contributions, thereby simplifying the prediction process. Bidirectional prediction is applied to the refined spectra, enabling the model to capture complementary fragmentation pathways: some molecules predominantly fragment in a forward direction, while others exhibit complex or reverse fragmentation behavior. By jointly learning both directions, the model can adaptively select the most appropriate fragmentation representation for each molecule.

Probabilistic mask. In the next step, the predicted spectra are processed by a probabilistic mask, composed of the following components: position-dependent probabilities, mass dependent scaling and neutral loss pattern.

Position-dependent probabilities learn which mass regions are generally more likely to contain peaks. It captures universal fragmentation patterns and provides foundation that works across different molecule types.

Mass-dependent scaling adjusts probabilities based on molecular weight. Depending on the size of molecules, it can make peaks more concentrated in lower *m*/*z* regions or have them spread across a wider range. This scaling considers the position of the peaks relative to the molecular mass rather than their absolute positions.

Neutral loss patterns account for commonly lost fragments during electron-induced fragmentation. For example, alcohols tend to lose $H_{2}O$, and amino acids often lose ${\mathrm{NH}}_{3}$. We explicitly incorporate these frequent neutral losses as a list of fragments and enhance their peaks with Gaussian boosts, slightly increasing the intensity of neighboring peaks. This adjustment accounts for measurement uncertainty and isotope patterns, while also smoothing gradients during model training. Gaussian parameters are predicted from graph embeddings using two shallow MLPs: one for the amplitude and one for the width (standard-deviation-like). Amplitudes are constrained to the (0, 1) range via a sigmoid, while widths are processed through a softplus function with a + 1 shift to prevent excessively small values. Gaussians are centered at the index corresponding to each neutral loss. The resulting Gaussians are not normalized, as normalization would cause a highly uncertain (wide) loss to contribute the same total mass as a precise one.

Overall, the mask system encodes the principle that certain fragmentation patterns are more common across different molecule types, while still allowing for molecule-specific variations. It combines learned chemical knowledge with adaptive prediction capabilities.

#### 3.3.4. Model Training

The target spectra used for model training are L1 normalized. This can be thought of as representing the spectra as discrete probability distributions. The loss function used for training is a combination of two components: reconstruction and retrieval loss. Reconstruction loss is inspired by the loss function used in [35]:

(2) $$\begin{array}{c} L_{\mathrm{rec}}=\frac{1}{B}\sum_{i=1}^{B}\frac{\sum_{k=1}^{D}{\left(m_{k}\left(\sqrt{t_{i,k}+\varepsilon }-\sqrt{{\tilde{p}}_{i,k}+\varepsilon }\right)\right)}^{2}}{\sum_{k=1}^{D}{\left(m_{k}\sqrt{t_{i,k}+\varepsilon }\right)}^{2}}, \end{array}$$

where ${\tilde{p}}_{i}$ are clamped predictions ${\tilde{p}}_{i}=\max\left(p_{i},0\right)$, with per sample predictions $p_{i}\in R^{D}$. Target spectra are denoted by $t_{i}\in R^{D}$, $m_{k}$ is the m/z value found a the *k*-th index and *B* is the batch size. ε is a small positive constant used to prevent errors like division by zero or issues arising from calculating the square root of zero. Normalizing the reconstruction loss only with the true spectra ensures that the magnitude of the loss is scaled appropriately for the complexity of the ground truth spectrum, without allowing the predicted spectrum’s own magnitude to interfere with the learning direction. This makes it a much safer and more robust choice for training than a loss that normalizes by both the predicted and true signals.

Retrieval loss component is a cross-entropy type of loss that is calculated between true and predicted spectra embeddings. Those embeddings are obtained through a small projection head (small MLP), that reduces dimensionality of the spectra from 500 to 128, projection head embeddings are L2 normalized. The projection head is trained together with the prediction model; however, it is affected only by retrieval loss, meaning it is decoupled and independent from reconstruction loss, and thus L1 normalized targets and L2 normalized embeddings do not interfere with each other. Let projected L2-normalized embeddings for predictions and targets be

(3) $$\begin{array}{cc} u_{i} & =\mathrm{proj}\left(p_{i}\right),\;v_{i}=\mathrm{proj}\left(t_{i}\right), \end{array}$$

with $∥u_{i}{∥}_{2}={∥v_{i}∥}_{2}=1$. Compute the score matrix $S\in R^{B\times B}$ whose element $s_{ij}$ is

(4) $$\begin{array}{c} s_{ij}=\frac{u_{i}\cdot v_{j}}{\tau }, \end{array}$$

where τ>0 is a hyperparameter. Cross-entropy retrieval loss uses the diagonal as positive pairs (index *i* matched to *i*). Let the label for query *i* be $y_{i}=i$. The per-batch retrieval loss is

(5) $$\begin{array}{c} L_{\mathrm{ret}}=-\frac{1}{B}\sum_{i=1}^{B}\log\frac{\exp(s_{ii})}{\sum_{j=1}^{B}\exp\left(s_{ij}\right)}=-\frac{1}{B}\sum_{i=1}^{B}\left(s_{ii}-\log\sum_{j=1}^{B}e^{s_{ij}}\right). \end{array}$$

The final loss function is a sum of reconstruction and retrieval loss

(6) $$\begin{array}{c} L_{\mathrm{total}}=L_{\mathrm{rec}}+\lambda \;L_{\mathrm{ret}}, \end{array}$$

with λ being a hyperparameter. Additionally, as a sanity check during training, a simple cosine similarity loss is computed for the test set. For a single sample *i*,

(7) $$\begin{array}{c} {\mathrm{sim}}_{i}=\frac{p_{i}\cdot t_{i}}{{∥p_{i}∥}_{2}\;{∥t_{i}∥}_{2}}. \end{array}$$

The loss per sample is $1-sim_{i}$. Batch loss

(8) $$\begin{array}{cc} L_{\cos} & =\frac{1}{B}\sum_{i=1}^{B}\left(1-{\mathrm{sim}}_{i}\right)=\frac{1}{B}\sum_{i=1}^{B}\left(1-\frac{p_{i}\cdot t_{i}}{{∥p_{i}∥}_{2}\;{∥t_{i}∥}_{2}}\right). \end{array}$$

A retrieval loss component is added to maximize recall values for library matching tasks. Moreover, it helps the model navigate angular dependencies better, stabilizing average cosine similarity values for the test set across different experiments. Reconstruction loss on its own has a tendency to overcompensate for differences in the direction of predicted vectors by adjusting their magnitudes, which can result in very low similarity values, even with good reconstruction loss values.

Model parameters are optimized using the Adam algorithm. We also employ a scheduler that reduces the optimizer learning rate on a loss plateau. The maximum number of training epochs is set to 500; however, an early stopping scheme is used, with patience parameter set to 50, which usually results in a number of training epochs no bigger than 350.

## 4. Conclusions

The proposed hybrid deep learning model successfully addresses the need to augment existing EI-MS spectral libraries by high-quality synthetic spectra. Trained on NIST14 (≤500 Da) and evaluated across ten random seeds, the model achieves strong library-retrieval performance (Recall@10 ≈ 80.8%) and an average raw cosine similarity of ≈0.74, indicating its capacity to generate spectra suitable for augmenting reference libraries. Moreover, ablation studies further confirm the positive influence of all major architecture components, although the bidirectional prediction module exhibited the highest variance.

However, the observed drop-off in Recall@1 (25%) suggests that model generalization remains biased toward molecules structurally similar to those in the training set, indicating an important direction for future improvement. We attribute the remaining errors to the dataset bias, limited spectral uniqueness, and inherent model’s approximations (e.g., integer *m*/*z* resolution and mass cutoff), suggesting that these are the main constraints to better identification performance.

Potential future work will focus on improving generalization and library matching tasks. Those could be achieved through a combination of data-centric, representation-level, and training-strategy enhancements. Increasing the data volume, for instance by incorporating newer versions of NIST spectral library, is likely to improve overall model performance, as neural network-based models are known to depend strongly on both the size and diversity of training data. Richer molecular representations could provide more expressive conditioning signals for spectrum prediction. Pretraining the encoders on large-scale self-supervised tasks may further improve robustness when fine-tuned on spectral data. During the training, more sophisticated regularization strategies such as peak-sensitive dropout could lead to higher embedding uniqueness. Finally, applying contrastive or metric learning to spectral embeddings could plausibly result in more effective grouping and the separation of molecular embeddings, thereby improving the model’s discrimination and generalization.

In practice, our approach provides a scalable complement and alternative to computationally expensive quantum-chemical simulations, partially alleviating gaps in library coverage. Future work will focus on further enhancing spectral uniqueness, increasing dataset diversity, and incorporating hybrid physics-informed features to improve model generalization, using the methods described above.

## Figures and Tables

**Table 1:** Average values of metrics for baseline model and ablation studies for the training regimen.

Model Variation	Training Loss	Test Loss	Test Cosine Similarity	Recall@10	Recall@5	Recall@1
baseline	0.403±0.039	0.417±0.026	0.659±0.049	0.734±0.063	0.654±0.069	0.392±0.057
disable_attention	0.512±0.076	0.480±0.036	0.444±0.021	0.599±0.089	0.511±0.089	0.271±0.063
replace_resnet_with_linear	0.503±0.032	0.446±0.015	0.616±0.032	0.715±0.028	0.625±0.030	0.360±0.023
fix_alpha_forward	0.424±0.011	0.452±0.003	0.676±0.005	0.720±0.009	0.632±0.011	0.358±0.011
replace_gnn_with_pool_mlp	0.663±0.033	0.600±0.004	0.437±0.016	0.370±0.009	0.286±0.010	0.125±0.006
disable_mask	0.464±0.059	0.448±0.030	0.366±0.021	0.672±0.072	0.583±0.074	0.322±0.053

**Table 2:** Summary of Recall@k and cosine similarity values for our model, NEIMS and RASSP across different replib splits.

Metric	HYBRID	NEIMS	RASSP SubsetNet
RASSP split replib			
Recall@1	24.0%	21.8%	58.9%
Recall@5	66.6%	59.6%	85.9%
Recall@10	80.6%	74.1%	90.9%
Full replib			
Recall@1	25.0%	22.9%	-
Recall@5	67.0%	61.3%	-
Recall@10	80.8%	75.3%	-
Small molecules, RS replib\full replib			
Recall@1	19.0%\19.3%	17.1%\17.6%	59.5%\-
Recall@5	62.0%\62.1%	54.0%\54.6%	89.0%\-
Recall@10	79.0%\79.0%	70.8%\71.3%	93.8%\-
Medium molecules, RS replib\full replib			
Recall@1	28.8%\28.6%	25.5%\26.2%	62.3%\-
Recall@5	72.2%\72.1%	65.3%\66.7%	87.5%\-
Recall@10	84.0%\83.7%	78.1%\79.0%	91.8%\-
Large molecules, RS replib\full replib			
Recall@1	26.3%\27.1%	29.1%\28.9%	33.5%\-
Recall@5	61.2%\65.0%	60.3%\63.8%	57.7%\-
Recall@10	71.5%\76.5%	70.1%\74.7%	67.1%\-
Average cosine similarities of predictions *			
Overall	0.74	0.76	0.88
Small molecules	0.77	0.78	0.91
Medium molecules	0.74	0.76	0.87
Large molecules	0.64	0.70	0.68

* For their respective replib splits.

**Table 3:** Properties used as atom and bond features.

Feature Type	Description	Size
Atom Features		
Atomic Number	Atom Number of an atom	61
Node Degree	Number of adjacent atoms	7
Valence	Number of explicit valences	7
Formal Charge	Integer electronic charge	5
Radical Electrons	Number of radical electrons	1
Hybridization	Type of hybridization	6
Aromaticity	Whether an atom is a member of an aromatic ring	1
Hydrogen	Number of hydrogens	6
Bond Features		
Bond Type	Single, double, triple, and aromatic	4
Conjugation	Is bond conjugated	1
Is in Ring	Is bond in a ring	1
Chirality	Stereo configuration	4

**Table 4:** Model and training hyperparameters and their values.

Hyperparameter	Value
MPNN encoder number of layers	3
MPNN encoder hidden layers dimension	256
MPNN encoder dropout probability	0.25
ResNet decoder input dimension	256
Number of ResNet blocks	1
ResNet block number of hidden layers	1
ResNet block hidden layers dimension	1024
Dropout probability inside ResNet block	0.2
ResNet decoder first head layer width	512
ResNet decoder second head layer width	256
ResNet decoder output dimension	500
Number of heads in cross-attention	4
Query, Keys and Values projection dimension	256
Projection head τ	0.04
Projection head hidden layers dimension	256
Projection head projection dimension	128
Total loss function λ	0.5
Batch size	256
Learning rate	0.001
Epochs	500
Patience	50
Optimizer	Adam
Scheduler	ReduceLROnPlateau (factor = 0.1, patience = 5)

## Data Availability

Training performance, dataset classification and prediction outputs of Hybrid Deep Learning Model for EI-MS prediction are publicly available in the Bridge of Knowledge Open Data Repository: https://doi.org/10.34808/hfda-sy14. The source code for this work can be available from the authors upon reasonable request.
